# Assessing the preparedness of primary healthcare facilities for the implementation of universal test and treat in uThukela District, South Africa: a cross-sectional study

**DOI:** 10.1136/bmjph-2025-002605

**Published:** 2025-10-15

**Authors:** Eunice Turawa, Wisdom Basera, Mbuzeleni Hlongwa, Thubelihle Mathole, Debbie Bradshaw, Edward Nicol

**Affiliations:** 1 Burden of Disease Research Unit, South African Medical Research Council, Cape Town, Western Cape, South Africa; 2 Department of Global Health, Faculty of Medicine and Health Sciences, Stellenbosch University, Cape Town, Western Cape, South Africa; 3 Public Health, Societies and Belonging, Human Sciences Research Council, Pretoria, Guateng, South Africa; 4 School of Nursing and Public Health, University of KwaZulu-Natal, Durban, KwaZulu-Natal, South Africa; 5 School of Public Health, University of Western Cape, Cape Town, Western Cape, South Africa

**Keywords:** Public Health, HIV, Cross-Sectional Studies

## Abstract

**Background:**

The availability of HIV guidelines, infrastructure, essential medicines and diagnostic tests for HIV services is a prerequisite to effective universal test-and-treat (UTT) services. However, evidence on public facility readiness for UTT services in rural, high HIV prevalence areas remain limited. This study provides a snapshot of facility readiness for UTT services in selected public health facilities within the uThukela district, KwaZulu-Natal, South Africa.

**Methods:**

A cross-sectional survey was conducted at 15 healthcare facilities in the uThukela district, KwaZulu-Natal Province, South Africa. Data were collected from public primary healthcare points, including three mobile clinics, three gateway clinics and nine Outpatient Departments, Community Health Centres and Hospitals (OPD-CHC-H). Questionnaires were programmed into Research Electronic Data Capture. Analysis was performed using STATA Software V.18, and results were summarised in percentages (%) and presented in tables and graph.

**Results:**

The study highlights gaps in UTT services in uThukela district, with variability in infrastructure availability across health facility types. Mean readiness was 86% (95% CI: 77.6% to 92.1%) for OPD-CHC-H; 70% (95% CI: 60.0% to 78.7) for gateway clinics and 67% (95% CI: 56.8% to 76.0%) for mobile clinics. Mean availability of indicators for basic amenities was 61% (95% CI: 50.7% to 70.5%); 90.4% (95% CI: 82.3% to 95.0%); and 80.9% (95% CI: 70.8% to 87.3%) in mobile clinics, gateway clinics, and OPD-CHC-H, respectively. HIV test kits, palliative care guidelines and improved water sources was limited across all facility types. Overall, facility readiness was 74% (95% CI: 64.2% to 82.2%), which was deemed satisfactory according to WHO-Service Availability and Readiness Assessment criteria.

**Conclusions:**

Persistent shortages in antiretrovirals, HIV test kits, palliative care guidance and logistics/operations hinder care; integrating HIV testing into routine screenings, expanding self-testing and adopting digital and personalised care models can ease burdens and improve outcomesCite Now

**WHAT IS ALREADY KNOWN ON THIS TOPIC**

- South Africa has implemented universal test-and-treat (UTT) in all public healthcare facilities nationwide.

**WHAT THIS STUDY ADDS**

- This study contributes to comprehensive knowledge on UTT implementation. uThukela’s district readiness is satisfactory, but antiretroviral (ARV) and HIV test-kit supplies, palliative-care guidance, essential amenities and community models (eg, HIV self-testing) require scale-up.

**HOW THIS STUDY MIGHT AFFECT RESEARCH, PRACTICE OR POLICY**

- These findings can help improve HIV policies, prioritise ARV drug and HIV test-kit supply. Update HIV palliative care guidance, scale up community-based services and refine readiness assessment tools to better guide policy and service delivery.

## Background

 HIV remains a significant public health concern, affecting about 39.9 million people worldwide.^1^ In 2023, about 39% fewer people acquired HIV compared to 2010, and sub-Saharan Africa achieved a 56% decline in new HIV incidence.^2^ Nonetheless, an estimated 1.3 million new infections occurred in 2023. Worldwide, approximately 31 million people currently access antiretroviral (ARV) treatment.^2^ About 9.3 million people living with HIV (PLHIV) are without treatment in 2023.^2^ About 86% of PLHIV knew their HIV status, 89% of those who knew their HIV status were on ARV treatment and 93% of those on treatment achieved viral suppression.^1^

South Africa has made noteworthy progress in its HIV treatment response, particularly through the rollout of antiretroviral therapy (ART), which has significantly reduced the national prevalence of HIV by 1.3% in 2022, and lowered the number of PLHIV to about 7.8 million from 7.9 million in 2017.^3^ Nearly 90% of the people knew their HIV status, with 91% of those on ART and 94% achieving viral suppression.^3^ Nonetheless, significant gaps in accessing HIV care and services remain, with notable differences between rural and urban areas.^4^ A substantial proportion of adolescents and young adults are still unaware of their HIV status or are not receiving ARV treatment following HIV diagnosis, and their viral loads remain unsuppressed.^5^

The WHO-recommended universal test and treat (UTT) policy advocates for early screening of all populations at risk of HIV infection and prompt initiation of ART by individuals diagnosed as HIV-positive, regardless of their CD4 count and clinical status.^6^ To improve viral suppression, same-day initiation (SDI) of ART (rapid ART) for individuals diagnosed with HIV was recommended.^7^ Many countries, including South Africa, have adopted and integrated this policy into their healthcare systems as early as 2016 to accelerate HIV management and initiation of treatment.^8^ This global synergy intends to halt the HIV epidemic by 2030. Heightened by the 95-95-95 targets, which proposed that 95% of PLHIV should be diagnosed, and 95% of those diagnosed should be on ART, and 95% of those on treatment should be virally suppressed.^9^ The UTT improves health outcomes by preventing HIV progression and increasing treatment coverage. UTT has been found to be cost-effective, supporting HIV epidemic control and strengthening healthcare systems.^2 10^ Previous studies highlighted the benefits of UTT services. The HPTN 052 randomised trial identified UTT as key to combating the HIV/AIDS epidemic.^11^ The Botswana Combination Prevention Project significantly reduces HIV incidence by implementing UTT services.^12^ These findings underscore the potential benefits of UTT services.

In many countries, implementing the UTT policy faced challenges due to the inadequate preparedness of health facilities. Only 5.9% of health facilities in Nepal were ready to offer HIV testing and counselling.^13^ Similarly, Songo *et al* discussed the findings of SHAPE studies in Malawi and Tanzania, highlighting the importance of a well-trained healthcare workforce. Increased patient loads for UTT services, without a corresponding rise in health provider capacity and inadequate support, raise concerns about the quality of care.^14^

In South Africa, primary healthcare (PHC) facilities deliver UTT services using a differential model that incorporates both facility-based and community-based approaches, such as mobile service points. Highly qualified healthcare providers typically lead the facility-based model, providing services such as adherence support and ARV collection. Meanwhile, the community-based model, led by nurses, delivers clinical care and medications at off-site service points.

A 2017 audit found that, on average, the PHC facilities performed worse than hospitals in terms of the availability of essential medicines and supplies, health workers staffing and physical infrastructure. Such inadequacy could result in low performance and poor satisfaction.^15^ Inadequate infrastructure and health system challenges fuel poor policy implementation.^16 17^ It is unclear whether the health facilities in the uThukela district are well-equipped to offer UTT services for the timely achievement of the Joint United Nations Programme on HIV/AIDS 2030 targets.^18^ In light of the substantial HIV burden and prevailing health system challenges in the country, this study seeks to evaluate the preparedness of PHC facilities to deliver UTT services within the uThukela district. Furthermore, it aims to identify context-specific strategies to improve the implementation and effectiveness of UTT service delivery in this rural setting. The findings from this study are expected to inform policy and contribute to the development of evidence-based guidelines to strengthen UTT services in underserved and rural communities^13 19^

## Methods

We conducted a facility-based, descriptive cross-sectional survey in the uThukela district, KwaZulu-Natal (KZN) Province, South Africa, to evaluate health facility’s readiness to offer UTT services. We employed a stratified sampling approach focusing on PHC facilities, which were selected because they are the key service points for HIV care and management in the district.

### Study setting and sampling

The study setting has been previously described extensively in a peer-reviewed publication.^20^ The uThukela district is a rural community with a high HIV burden (27.9%).^21^ About 95% of the residents are without medical insurance,^22^ offering an ideal setting to monitor UTT services. The uThukela district is the fourth-largest municipality in the KZN province, with a population of 789 092, according to the 2022 census data. The district, comprising three local municipalities (LMs), was recently established by merging five previous LMs following the August 2016 Municipal elections. Among the LMs, Alfred Duma has the largest population (50.8%), followed by Inkosi Langalibalele (29.4%), and Okhahlamba (19.8%).^23^ The study assessed the PHC facility’s readiness to provide UTT services in a high-HIV-prevalence district. The health facilities comprise mobile clinics, gateway clinics, Community Health Centres (CHC), fixed PHC clinics, community day centres (CDCs), mobile units and outreach centres managed by the Health Systems Trust.^24^ Of the 56 public health facilities providing HIV services, including ART, in the uThukela district, 18 PHC facilities were purposively selected for the main study using simple random sampling. Selection was based on each facility’s average number of positive HIV tests per month, with priority given to those with higher HIV positivity rates and monthly HIV testing volumes exceeding 4000 clients. At the time of data collection, 15 of the selected facilities were actively providing HIV services and were therefore included in the final study sample. Table 1 provides a list of selected health facilities per municipality.

**Table 1:** Health facility selection process for UTT service readiness assessment in uThukela district, South Africa

Local municipality	Name of health facilities	Type of facility
Alfred Duma Municipality	Ladysmith Gateway Clinic	Gateway Clinic
	Limehill Clinic	Clinic
	Outer West Mobile	Mobile Outlet
	St Chads (CHC)	CHC
	Walton Central Clinic	Clinic
	Steadville Clinic	Clinic
	Isigweje	Clinic
Okhahlamba Municipality	Emmaus Hospital (OPD)	OPD
	Bergville Clinic	Clinic
	Emmaus Gateway Clinic	Gateway Clinic
	Bergville mobile 2	Mobile Outlet
	Bergville Mobile 3	Mobile Outlet
Inkosi Langalibalele Municipality	Wembezi clinic	Clinic
	Estcourt Gateway Clinic	Gateway Clinic
	Injisuthi clinic	Clinic

CHC, community health centre; OPD, outpatient department; UTT, universal test-and-treat.

### Study participants

The study participants included facility managers (FMs), operation managers (OMs) and key personnel in health service operations in the selected facilities. Participants were contacted and recruited and interviewed on health facility’s preparedness to offer UTT services.

### Data collection and management

We collected data on the general readiness of health facilities to offer UTT services in the rural community of the uThukela district. Service readiness was defined as ‘the capacity of the health facility to provide UTT services to the community’. It measures the availability of equipment and supplies necessary to provide services across five domains.^25^ Table 2 presents tracer indicators measured in each domain. The Service Availability and Readiness Assessment (SARA) is a survey tool jointly developed by the WHO and UNAIDS to generate tracer indicators for assessing the availability and readiness of health facilities to provide healthcare services.^25^ The methodology combined identified best practices with lessons from countries’ health facility assessments and specific HIV/AIDS programme evaluation processes. It effectively assesses infrastructure and equipment for healthcare service readiness.

**Table 2:** Assessment domains and tracer indicators for UTT service readiness in uThukela district, KZN, South Africa

Domains	Tracer indicators		Domain score (n/N)×100
Basic amenities (7 items)	Functional computers Consulting room with privacy Improved water source in the facility	Modern toilets for clients Emergency transport Secondary power supply source Power supply (central grid)	(n/7)×100
Basic equipment (4 items)	HIV rapid test kits ART data register and tools	Stationery supply±2 weeks TB screening equipment	(n/4)×100
Standard infection prevention and control (10 items)	Hand washing soap Sharps container (safety box) Infectious waste-safe disposal Infection control guidelines Environmental disinfectant	Waste bins with a lid Disposable syringes Auto-disable syringes Clean running water/pipe Alcohol-based hand disinfectant.	(n/10)×100
HIV guidelines (4 items)	National ART guidelines Palliative care guideline	National HIV/AIDS management guidelines HIV VCT guidelines	(n/4)×100
Essential ARV drugs (24 items)	Abacavir Cotrimoxazole Didanosine Efavirenz Lamivudine Lamivudine abacavir Nevirapine Ritonavir Stavudine syrup Tenofovir disoproxil fumarate Tenofovir disoproxil+lamiv+efavirenz Zidovudine_lamivudine	Efavirenz syrup Emtricitabine Enfuvirtide Lamivudine syrup Nevirapine syrup Stavudine lamivudine Stavudine lamivudine -nevi Tenofovir+Emtricitabine+ Efav Tenofovir-emtricitabine Zidovudine+lamivudine Zidovudine+lamivudine Zidovudine syrup.	(n/24)×100
Overall service readiness			Mean score of all domains: (a+b+c+d+e)/5.

ART, antiretroviral therapy; ARV, antiretroviral; KZN, KwaZulu-Natal ; TB, tuberculosis; UTT, universal test-and-treat; VCT, Voluntary HIV Counselling and Testing.

The SARA tool has previously been adapted and used in different settings to assess facility readiness.^13 26 27^ For the current survey, the tool was adapted to suit the specific objectives and context of this study. Relevant sections were selected based on the study focus, and the tool was contextualised to align with national health system structures and terminology. Additional context-specific items were included to capture locally relevant information. The requirements for UTT service in South African HIV management cascade were classified into five domains of UTT service readiness: (1) basic amenities, (2) basic equipment, (3) standard infection prevention and control, (4) HIV care and management guidelines and (5) essential medicines and commodities^25^ as outlined in table 2.

To ensure clarity and feasibility, the tool was piloted in selected facilities, which were then excluded from the main study. Field workers received training on the use of the tool, and standard operating procedures (SOPs) were followed to ensure consistency. The original WHO-SARA tool was duly referenced in the manuscript.

Health FMs or senior healthcare professionals were contacted to schedule interviews regarding UTT service readiness. Facility inventory-validated questionnaires were programmed into Research Electronic Data Capture and used to assess the availability of basic amenities and essential equipment for UTT services, including HIV test kits, treatment guidelines and essential medicines in the surveyed health facilities. Follow-up questions were asked to clarify uncertainties when items were not visibly displayed. The data were anonymised and cleaned prior to analysis.

### Data analyses

We adapted the WHO-SARA tool to assess health facility readiness for offering UTT services in selected facilities of the uThukela district. All included service points operate at the PHC level. We categorised health facilities into three types: Mobile clinics, Gateway clinics and Outpatient Departments (OPD), CHC and Day Hospitals (OPD-CHC-H) based on facility type and designated services. This stratum was necessary to differentiate between facility structures and the required tracer indicators for HIV services.^28^ Considering the high burden and public health impact of HIV in the country, South Africa has a unique and broad HIV management cascade, including essential medicines. Therefore, we used local essential medicine list and commodities, which were not necessarily the SARA list, for the current assessment.^29^

For each domain, we coded the presence or absence of tracer indicators as ‘available’ (√) or ‘not available’ (*), report not seen (**), never available (***) or not applicable (n/a). To measure service readiness, we calculated the proportion of available tracer indicators across included health facilities and domains, reported them as the proportion (n/N) with the tracer indicator present on the assessment day. We stratified by facility type, and readiness scores for each domain were calculated by dividing the available tracer indicators by the total required indicators, expressed as a percentage (%).

We calculate the Readiness Index (RI) composite score to assess the overall readiness of the facility to offer UTT services. Data were analysed using STATA Software V.18.0 (StataCorp). Results were summarised narratively using tables (proportions and percentages (%)) and figure. We tested for normality in the data using the Shapiro-Wilk test, which yielded a p value of 0.277, more significant than 0.05, suggesting that the data are normally distributed. Levene’s test was used to check the homogeneity of variances, which shows that variances were homogenous across health facility categories with p values <0.645, >0.708 and >0.354 for mobile clinics, gateway clinics and OPD-CHC-H, respectively. Due to variability in required tracer indicators per facility category (eg, mobile clinics do not need a secondary power supply), direct comparison between facility categories to determine differences in performance was not feasible. However, we described an overview of facility readiness to offer UTT services in the surveyed facilities.

## Results

### Characteristics of healthcare facilities

We included 15 public health facilities in the uThukela district, KZN province, South Africa, which comprise three mobile clinics (20%), three gateway clinics (20%), and nine OPD-CHC-H (60%). The findings were stratified by facility type, readiness domain and tracer indicators, as shown in table 3. We summarised the mean health facilities with tracer indicators on the day of the interview.

**Table 3:** The proportion of health facility types with tracer indicators by domain in uThukela district, KZN, South Africa

Tracer indicators per domain	Mobile clinics N=3	Gateway clinics N=3	OPD-CHC-H N=9	Total facilities with items N=15
**Basic amenities**	**n (%)**			**n/N (%)**
Emergency transport	1 (33.3)	3 (100.0)	6 (66)	10/15 (67)
Functional computers	3 (100.0)	3 (100.0)	8 (88)	14/15 (93)
Improved water source within facilities	2 (66.6)	3 (100.0)	4 (44)	9/15 (60)
Modern toilets for clients	2 (66.6)	3 (100.0)	9 (100.0)	14/15 (93)
Power supply (central grid)	3 (100.0)	3 (100.0)	9 (100.0)	15/15 (100)
Consulting rooms with privacy	*	2 (66.6)	8 (88)	10/15 (67)
Secondary power supply source	n/a	2 (66.6)	7 (77)	9/12 (80)
Domain score	61.0%	90.4%	80.9%	77.4%
Basic equipment				
ART data registers and tools	3 (100.0)	3 (100.0)	9 (100.0)	15/15 (100)
HIV test kits	2 (66.6)	2 (66.6)	8 (88)	12/15 (80)
Stationery supply	3 (100.0)	3 (100.0)	9 (100.0)	15/15 (100)
TB screening tools	3 (100.0)	3 (100.0)	9 (100.0)	15/15 (100)
Domain score	91.6%	83.3%	97.2%	90.7%
Standard infection prevention and control				
Alcohol-based hand disinfectant	3 (100.0)	2 (66.6)	9 (100.0)	14/15 (93)
Auto syringes	0 (0)	**	6 (66)	6/15 (40)
Clean running water or pipe	2 (66.6)	3 (100.0)	8 (88)	13/15 (87)
Disposable gloves	3 (100.0)	2 (66.6)	9 (100.0)	14/15 (93)
Disposable syringes	3 (100.0)	2 (66.6)	9 (100.0)	14/15 (93)
Environmental disinfectant	3 (100.0)	2 (66.6)	9 (100.0)	14/15 (93)
Hand washing soap	1 (33.3)	2 (66.6)	9 (100.0)	12/15 (80)
Infection control guidelines	2 (66.6)	3 (100.0)	9 (100.0)	14/15 (93)
Sharps containers	3 (100.0)	2 (66.6)	9 (100.0)	14/15 (93)
Waste bins with lids	3 (100.0)	2 (66.6)	9 (100.0)	14/15 (93)
Domain score	76.6%	66.6%	95.5%	79.6%
HIV management guidelines				
HIV/AIDS management guidelines	1 (33.3)	2 (66.6)	9 (100.0)	12/15 (80)
National ART guidelines	1 (33.3)	2 (66.6)	9 (100.0)	12/15 (80)
National HCT guidelines	2 (66.6)	3 (100.0)	9 (100.0)	14/15 (93)
Palliative care guidelines	0 (0)	1 (33.3)	5 (55)	6/15 (40)
Domain score	33.3%	66.6%	88.8%	62.9%
Essential medicine and commodity				
Abacavir	3 (100.0)	1 (33.3)	9 (100.0)	13/15 (87)
Co-trimoxazole	3 (100.0)	3 (100.0)	9 (100.0)	15/15 (100)
Didanosine	3 (100.0)	1 (33.3)	9 (100.0)	13/15 (87)
Efavirenz	3 (100.0)	2 (66.6)	9 (100.0)	14/15 (93)
Efavirenz syrup	2 (66.6)	*	*	2/15 (13)
Emtricitabine	2 (66.6)	2 (66.6)	6 (66)	10/15 (67)
Enfuvirtide	1 (33.3)	*	*	1/15 (6.6)
Lamivudine	3 (100.0)	1 (33.3)	9 (100.0)	13/15 (87)
Lamivudine abacavir	3 (100.0)	2 (66.6)	7 (77.7)	12/15 (80)
Lamivudine syrup	2 (66.6)	2 (66.6)	9 (100.0)	13/15 (87)
Nevirapine	3 (100.0)	2 (66.6)	9 (100.0)	14/15 (93)
Nevirapine syrup	3 (100.0)	2 (66.6)	9 (100.0)	14/15 (93)
Ritonavir	*	2 (66.6)	2 (22)	4/15 (26.6)
Stavudine lamivudine	1 (33.3)	*	*	1/15 (6.6)
Stavudine lamivudine+nevi	1 (33.3)	*	*	1/15 (6.6)
Stavudine syrup	*	*	3 (33)	3/15 (20)
Tenofovir+emtricitabine+efavirenz	3 (100.0)	2 (66.6)	9 (100.0)	14/15 (93)
Tenofovir disoproxil fumarate	2 (66.6)	*	8 (88)	10/15 (67)
Tenofovir disoproxil+lamiv.+ efavirenz	2 (66.6)	1 (33.3)	3 (33)	6/15 (40)
Tenofovir+emtricitabine	3 (100.0)	3 (100.0)	8 (88)	14/15 (93)
Zidovudine+lamivudine	3 (100.0)	*	3 (33)	6/15 (40)
Zidovudine+lamivudine	1 (33.3)	*	2 (22.2)	3/15 (20)
Zidovudine syrup	2 (66.6)	1 (33.3)	9 (100.0)	12/15 (80)
Zidovudine+lamivudine	3 (100.0)	2 (66.6)	9 (100.0)	14/15 (93)
Domain score	72.2%	42.2%	65.2%	61%

* Not reported

ART, antiretroviral therapy; HCT, HIV counselling and testing; KZN, KwaZulu-Natal; n/a, not available; OPD-CHC-H, Outpatient Departments, Community Health Centres and Hospitals.

### Availability of basic amenities in surveyed facilities

Table 3 shows the tracer indicators available per facility type. Basic amenities assessed include central grid power supply, emergency vehicle/ambulance (e.g, the facility has a functioning ambulance/emergency vehicle on-site or shared with a nearby facility, with fuel available on the day of the survey), secondary power sources, improved water sources, consultation room with audio and visual privacy, modern patient toilets and functional computers. Most health facilities have required indicators. The most frequently available tracer indicators are functional computers, modern toilets and a central power supply. However, improved water sources (ie, improved water source in the facility/ water from a public tap/standby pipe/ borehole) were the least available items.

Improved water sources were available in 44% (95% CI: 34.0% to 54.2%) of OPD-CHC-H category. Only a third (33.3% (95% CI: 23.9% to 43.1%)) of mobile clinics had emergency vehicles/ambulances to transport patients to hospitals in the event of an emergency. The gateway clinics had all items except a consulting room with audio and visual privacy and a secondary power source in two-thirds of the gateway clinics. The average availability of tracer indicators for basic amenities was 61% (95% CI: 50.7% to 70.5%); 90.4% (95% CI: 82.3% to 95.0%) and 80.9% (95% CI: 70.8% to 87.3%) in mobile clinics, gateway clinics and OPD-CHC-H health facilities, respectively.

### Basic equipment required for UTT services

The presence of tracer indicators, including HIV rapid test kits, tuberculosis screening tools for HIV disease, ART data registers and tools, and stationery supplies, was assessed. Table 3 shows the proportion of health facilities with tracer indicators on the survey day. All tracer indicators were available in OPD-CHC-H, except at one facility that lacked HIV rapid test kits. There is a slight variation in the mean availability of indicators between health facility categories. Mean availability of tracer indicators was 97.2% (95% CI: 91.4% to 99.3%), 83.3% (95% CI: 74.1% to 89.7%) and 91.6% (95% CI: 83.6% to 95.8%) for OPD-CHC-H, gateway clinics and mobile clinics, respectively. Among health facility types, HIV rapid test kits were the least available item.

### Standard infection prevention and control

Health facility readiness was assessed based on the availability of tracer indicators for prevention and control of infection in HIV services. Items assessed include alcohol-based disinfectants, auto-syringes, a clean running water supply, disposable gloves, disposable syringes, environmental disinfectants, handwashing soap, infection control guidelines, sharp containers and waste bins with lids. All listed items were reported and observed in 95.5% (95% CI: 88.7% to 98.3%) of the OPD-CHC-H facilities, 66.6% (95% CI: 55.8% to 75.1%) of the gateway clinics and 76.6% (95% CI: 66.4% to 83.9%) of the mobile clinics. A third, 33% (95% CI: 23.9% to 43.1%) of mobile clinics had handwashing soap available. Clean running water was absent in one OPD-CHC-H facility and mobile clinic. All gateway clinics had access to clean running water and displayed infection control guidelines in the facilities. Table 3 shows the proportion of health facilities with tracer indicators.

### Availability of HIV/AIDS guidelines

Table 3 shows the availability of HIV/AIDS guidelines for UTT services. Indicators assessed include national ART guidelines, HIV counselling and testing protocols (HCT), palliative care guidelines and HIV clinical management. Indicator availability varied significantly. The national HCT guidelines were the most available item across health facilities, with an average availability of 88.8% (95% CI: 79.9% to 93.6%) in OPD-CHC-H, 66.6% (95% CI: 55.8% to 75.1%) in gateway clinics and 33.3% (95% CI: 23.9% to 43.1%) in mobile clinics. Palliative care guidelines were the least available, observed in 55.5% (95% CI: 44.7% to 64.9%) of OPD-CHC-H and 33.3% (95% CI: 23.9% to 43.1%) of gateway clinics, but were unavailable in mobile clinics.

### Availability of essential medicines and commodities

The availability of essential medicines for HIV treatment was assessed. Table 3 displays the available medicines with valid expiration dates. Most listed medicines were available on the day of the survey. Mobile clinics had most of the required essential medicines compared with other health facilities. Across health facilities, available medicines include cotrimoxazole, efavirenz, nevirapine tablet and syrup, lamivudine and abacavir, tenofovir+emtricitabine+efavirenz, ritonavir tenofovir+emtricitabine and zidovudine+lamivudine. The mean indicator for essential medicines was 72.2% (95% CI: 62.1% to 80.5%), 42.2% (95% CI: 32.1% to 52.2%) and 65.2% (95% CI: 54.8% to 74.2%) for mobile clinics, gateway clinics and OPD-CHC-H, respectively.

### Domain readiness per health facility category

Table 3 shows health facility readiness by facility type and assessed domains. The OPD-CHC-H appears to be well-prepared compared with others. Tracer indicators were present in most of the OPD-CHC-H facilities on the day of the assessment. Mobile clinics had the highest number of service points with essential medicines, at 72.2% (95% CI: 62.1% to 80.5%); however, HIV management guidelines were observed in only 33.3% (95% CI: 23.9% to 43.1%) of the clinics. Gateway clinics scored above 50% for most domains but lagged in essential medicine, at 42.2% (95% CI: 32.1% to 52.2%).

**Figure 1:**
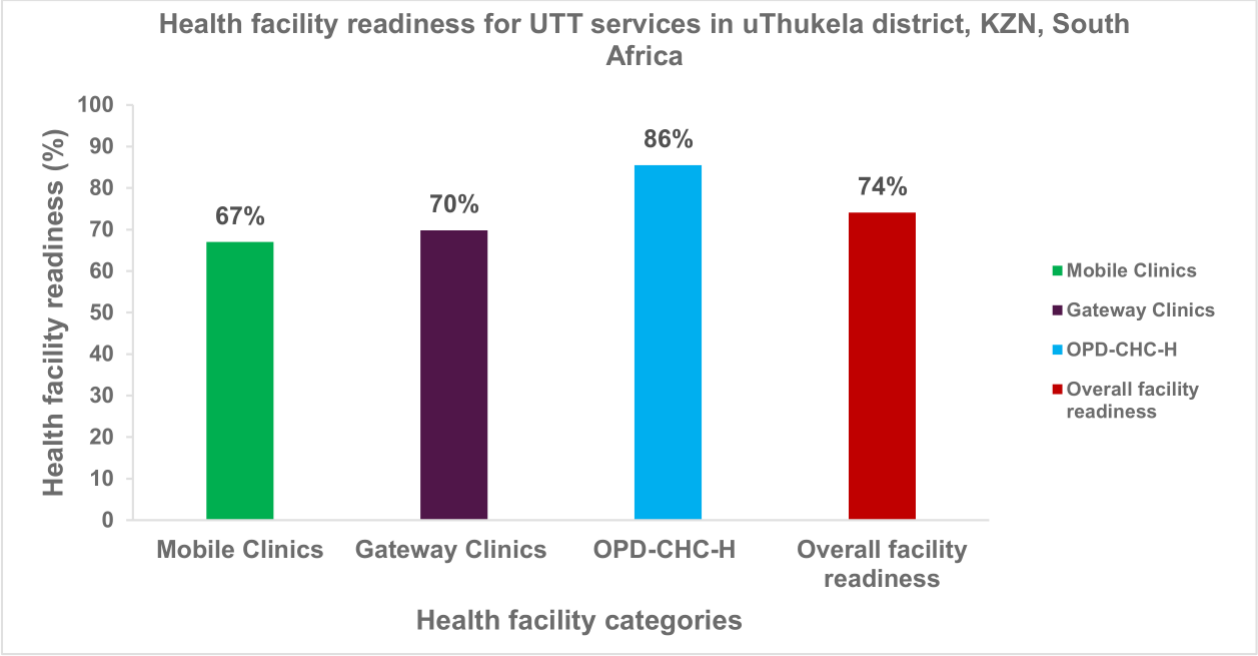
The overall facility readiness for UTT service in surveyed facilities in the uThukela district, KZN, South Africa. KZN, KwaZulu-Natal; OPD-CHC-H, Outpatient Departments, Community Health Centres and Hospitals; UTT, universal test-and-treat.

Figure 1 presents the overall readiness of health facilities to offer UTT services in the Thukela district. Readiness score was estimated at 67% (95% CI: 56.8% to 76.0%) for mobile clinics, followed by gateway clinics at 70% (95% CI: 60.0% to 78.7%). The OPD-CHC-H category had the highest score, at 86% (95% CI: 77.6% to 92.1%), preparedness for UTT services.

The overall readiness score was 74% (95% CI: 64.2% to 82.2%). Based on SARA-tool recommendations, ‘observation of at least one essential medicine with a valid expiration date in a grouped drug, or 29% of the health facilities that offer HIV care and support services’, qualifies a health facility for service readiness. The facilities achieved an overall readiness score of 74% (figure 1), reflecting satisfactory readiness for UTT service delivery.

## Discussion

UTT has improved HIV diagnosis and ART initiation, leading to significant improvement in patient experience and a reduction in HIV-related mortality.^30^

This survey assessed the readiness of PHC facilities to provide UTT services in a district with a high HIV prevalence and identified strategies for improvement. The key findings provide valuable benchmarks for monitoring the implementation and progress of the UTT policy.

Assessed health facilities were stratified by service scope. The mean overall readiness score across facilities was 74%, indicating satisfactory preparedness. However, important gaps were identified in mobile and gateway clinics. The current study proposes strategies to address these gaps, ensuring consistent and high-quality UTT service.

### Basic tracer indicators in UTT services

Mobile clinics had the lowest availability of basic amenities, lacking consultation rooms with audio and visual privacy. Additionally, the availability of improved water sources was low among OPD-CHC-H facilities. The absence of a private consulting room raises confidentiality concerns and may deter patients from seeking HIV testing and counselling in these health facilities due to perceived insecurity. Previous reports indicated that 79% of health facilities in KZN were characterised by insufficient resources, limited waiting areas, poor conditions and inadequate privacy.^31^ Mnyaka *et al* highlighted an acute shortage of resources in the implementation of UTT policy in the Eastern Cape province.^16^

Insufficient preparedness is common across the African continent. A study conducted in Ethiopia reported low facility readiness for intensive care services in PHC settings. Citing inadequate infrastructure, a lack of medical equipment and shortages of laboratory reagents as key barriers to health facility readiness.^32^ Despite modest improvements in ART adherence and viral suppression among HIV-infected youths in South Africa, those who remained in care 12 months postdiagnosis are below the UNAIDS 95-95-95 targets.^30^

Insufficient resources and health system weaknesses indirectly contribute to the global 9.3 million PLHIV without treatment.^33^ Adequate resources and supportive policy environment are vital for long-term HIV treatment engagement and best outcomes in HIV management.^34^

### Infection prevention and control

While many facilities were well-equipped with infection prevention and control items, a mobile clinic was without handwashing soap. There is a general shortage of auto-syringes across healthcare facilities.

An estimated 55% of OPD-CHC-H facilities had no reliable source of clean water, which may compromise care quality and increase health risks. This finding aligns with Beale *et al*, highlight that insufficient infection prevention and control in healthcare settings increases the risk of infection transmission and diarrhoea cases, which can further complicate HIV disease.^35^

Poor infection prevention and control continue to be a global issue. The UNICEF reported that one in four health facilities in Eastern and Southern Africa lacks clean water services.^36^ An adequate supply of clean water and handwashing soap is essential for achieving SDG target 3.8, equitable access to quality healthcare and improved disease outcomes.**^37^**

### Testing kits in UTT service

HIV rapid testing kits were unavailable in a subset of surveyed facilities, a shortfall associated with adverse effects on community HIV care.^38^ Mwisongo *et al* identified substandard testing practices, poor adherence to protocols, together with low rates of condom use among young people aged 15–24, as key hindrances to HIV in Africa.^2 39^

In Addis Ababa, one-quarter of public health facilities were without HIV test kits.^40^ Nicol *et al* attribute similar shortages to rising testing demand that is not matched by adequate supply.^41^ These gaps potentially delay HIV diagnosis and treatment and further undermine efforts towards the UNAIDS 2023 goal. Despite the high acceptability (84.7%) and improved performance of new self-testing technologies, these services remain scarce in the public health system.^42 43^ Implementing UTT services without sufficient diagnostic equipment in health facilities, particularly in rural communities, may widen the gaps in HIV services.

### Availability of HIV guidelines

The availability of palliative care guidelines to drive services in HIV management was consistently low across surveyed facilities. HIV treatment guidelines traditionally emphasise ART and the management of opportunistic infections, with limited attention given to palliative care, HIV testing, early ART initiation and linkage of PLHIV to care. Palliative care is vital for enhancing quality of life by relieving pain, managing symptoms and providing emotional, psychosocial and spiritual support, which can improve treatment adherence, patient outcomes and overall survival.^44^ Poor palliative care in HIV services is not unique to South Africa. Similar challenges, depriving patients of the full spectrum of care necessary for optimal health outcomes, have been reported in other African countries.^45^ Mnyaka *et al* reported a lack of SOPs for HIV care during UTT implementation in the Eastern Cape.^16^ Maluleka *et al* highlight the need for clear, frequently updated and widely circulated guidelines to ensure effective and standardised UTT service delivery.^17^ The provision of palliative care in HIV management is inadequate. Findings from community healthcare workers indicated that inadequate counselling, lack of patient education, weak support systems and persistent socioeconomic challenges lead to the use of alternative therapies in HIV care.^46^ However, HIV testing coverage has improved in South Africa.^41^

### Availability of essential medicines

ART significantly improves the quality of life and expectancy of PLHIV infection. Our findings showed improved ARV availability across the surveyed district. However, we identified an acute shortage of ARVs in some facilities. This observation aligns with Hwang *et al*’s report, which indicates that ARV stockouts are more common in African regions compared with other parts of the world.^47 48^ Between 2005 and 2014, about 35.7% of 1703 clinics across 35 countries experienced ARV stockouts.^48^

The lack of access to essential ARV medications poses a significant threat to the health and well-being of PLHIV. It interrupts treatment, which can lead to increased HIV transmission, morbidity and mortality and worsen mental health outcomes.^31 49^ These shortages undermine government efforts to control and eradicate HIV/AIDS, with additional burdens on individuals and healthcare systems. Conversely, Mnyaka *et al* noted that ARV stockouts rarely occur in the Eastern Cape.^16^ Ensuring timely access to ARVs is crucial for supporting UTT services and achieving the UNAIDS targets for the eradication of HIV/AIDS epidemics within the stipulated timeframe.

### Health system implications

Despite the progress in scaling up HIV testing and management in South Africa.^3^ This study highlights gaps in the implementation of UTT services in the surveyed district. Given the country’s high HIV prevalence, UTT services have profound implications for health systems and policies.

In addition, limited diagnostic equipment and insufficient ART supplies contribute to overcrowded clinics and extended waiting times, thereby reducing patient satisfaction, including the use of UTT services. Resource constraints compromise the quality of care. Inefficient ART supply chains result in treatment interruptions, compromised viral suppression rates and heightened risk of drug resistance, complicating HIV management and control.

To address these gaps, policy-makers may need to reallocate resources and prioritise investments in UTT infrastructure, including the provision of HIV test kits, improved water sources and palliative care guidelines to bridge the existing gaps. Without adequate resources, achieving the global UNAIDS 95-95-95 goals remains questionable.

Community-based approaches, such as self-testing, expanded differentiated care models and adoption of digital health solutions, may be areas to consider in UTT services. These shifts could maximise and enhance the effectiveness of existing resources while maintaining the accessibility and efficacy of UTT services.

Similarly, integrating HIV testing into general disease screening, implementing targeted interventions for high-risk populations, such as young males, and promoting preventative strategies like Pre-Exposure Prophylaxis (PrEP) to enhance the effectiveness of UTT services in the uThukela district may be necessary.

### Strengths and limitations of the study

This study is the first survey conducted in a resource-limited, high-HIV-prevalence district using an adapted version of the WHO-SARA tool to assess facility readiness for UTT services. The study identified potential barriers to implementing UTT services in the uThukela district and provided insights into areas that require improvement to ensure comprehensive UTT service delivery in rural communities.

However, there are notable limitations. Study scope was limited to a single district (uThukela district), with a relatively small sample size for the different facility types, which may limit the generalisability of the findings to the standard operations of fully functional public hospitals in other settings, as well as at the national level.

Larger sample sizes within facility categories could have provide a more comprehensive understanding of sub-district readiness. In addition, the reliability of organisational readiness assessment tools remains uncertain,^50 51^ and recommendations for focused questions to enhance tool usability and reliability have been suggested.^52^

This research did not consider the potential modifications in service delivery resulting from the COVID-19 pandemic, which may have influenced the operational readiness of facilities. Furthermore, we were unable to assess service availability comprehensively due to insufficient data. The temporary unavailability of tracer indicators on the day of the survey may have also biased the findings.

Future research should address these limitations by using a national sample to explore the relationship between resource availability and UTT implementation more broadly. Expanding the sample size and refining readiness assessment tools can enhance the reliability and applicability of the results, informing policy and practice.

## Conclusion and recommendation

This study highlights critical gaps in healthcare facility preparedness to deliver UTT services, primarily characterized by shortages in essential resources such as ARVs, HIV test kits and palliative care guidelines. These deficiencies underscore the need for strategic to strengthen healthcare systems, streamline supply chains and enhance resource availability, ensuring UTT services are widely accessible. The findings also highlight the importance of addressing logistical and operational challenges in UTT services, particularly in rural settings.

To enhance UTT service delivery, the government and stakeholders should prioritise investment in UTT infrastructure to ensure health facilities are adequately equipped to meet the demands of UTT services. This includes a steady supply of essential resources, such as ARVs, HIV test kits and updated palliative care guidelines, all of which are vital for effective treatment and patient support.

Expanding community-based approaches, such as self-testing and other models of care, can help overcome barriers like distance and limited access to formal healthcare settings. Similarly, differentiated care models tailored to individual patient needs, such as providing multimonth ARV prescriptions for stable patients, can reduce the burden on healthcare facilities while delivering more efficient and personalised care.

Lastly, adopting digital health solutions, including mobile healthcare platforms and digital health records, can enhance UTT service delivery. These technologies enhance communication, streamline data management, facilitate patient monitoring and facilitate the timely delivery of interventions and remote consultations, particularly in underserved areas. Addressing these challenges can improve the implementation and sustainability of UTT services, contribute to the global effort to reduce the burden of HIV/AIDS in line with the UNAIDS 95-95-95 targets.

## Data Availability

All data relevant to the study are included in the article or uploaded as supplementary information.
